# The role of *JrPPO*s in the browning of walnut explants

**DOI:** 10.1186/s12870-020-02768-8

**Published:** 2021-01-06

**Authors:** Shugang Zhao, Hongxia Wang, Kai Liu, Linqing Li, Jinbing Yang, Xiuhong An, Pingping Li, Linying Yun, Zhihua Zhang

**Affiliations:** 1 College of Life Sciences, Hebei Agricultural University, Baoding, 071001 China; 2 Mountainous Areas Research Institute of Hebei, Baoding, 071001 China; 3 National Engineering Reseach Center for Agriculture in North Mountainours areas, Baoding, 071001 China; 4 College of Horticulture, Hebei Agricultural University, Baoding, 071001 China

**Keywords:** Walnut, Explant, Vermiculite, Phenols, *JrPPO*

## Abstract

**Background:**

Tissue culture is an effective method for the rapid breeding of seedlings and improving production efficiency, but explant browning is a key limiting factor of walnut tissue culture. Specifically, the polymerization of PPO-derived quinones that cause explant browning of walnut is not well understood. This study investigated explants of ‘Zanmei’ walnut shoot apices cultured in agar (A) or vermiculite (V) media, and the survival percentage, changes in phenolic content, POD and PPO activity, and *JrPPO* expression in explants were studied to determine the role of PPO in the browning of walnut explants.

**Results:**

The results showed that the V media greatly reduced the death rate of explants, and 89.9 and 38.7% of the explants cultured in V media and A media survived, respectively. Compared with that of explants at 0 h, the PPO of explants cultured in A was highly active throughout the culture, but activity in those cultured in V remained low. The phenolic level of explants cultured in A increased significantly at 72 h but subsequently declined, and the content in the explants cultured in V increased to a high level only at 144 h. The POD in explants cultured in V showed high activity that did not cause browning. Gene expression assays showed that the expression of *JrPPO1* was downregulated in explants cultured in both A and V. However, the expression of *JrPPO2* was upregulated in explants cultured in A throughout the culture and upregulated in V at 144 h. *JrPPO* expression analyses in different tissues showed that *JrPPO1* was highly expressed in stems, young leaves, mature leaves, catkins, pistils, and hulls, and *JrPPO2* was highly expressed in mature leaves and pistils. Moreover, browning assays showed that both explants in A and leaf tissue exhibited high JrPPO2 activity.

**Conclusion:**

The rapid increase in phenolic content caused the browning and death of explants. V media delayed the rapid accumulation of phenolic compounds in walnut explants in the short term, which significantly decreased explants mortality. The results suggest that *JrPPO2* plays a key role in the oxidation of phenols in explants after branch injury.

**Supplementary Information:**

The online version contains supplementary material available at 10.1186/s12870-020-02768-8.

## Background

Walnut (*Juglans regia* L.), also known as Persian walnut, belongs to the Juglandaceae family and is native to Southeast Europe, Western Asia and Southwest China [1, 2]. Owing to its strong wood and nutritious nuts, this plant species is widely distributed in all parts of the world except Antarctica [3]. In recent years, with the development of improved cultivars, seedlings of excellent cultivars have become increasingly popular with farmers. However, the traditional low-efficiency method of grafting to generate seedlings has greatly limited the propagation and popularization of superior cultivars [4]. Over the past several years, tissue culture has become an effective method for rapid breeding of seedlings and improving production efficiency [5]. Nevertheless, explant browning is one of the key factors limiting tissue culture of walnut [6, 7]. Various methods have been used to reduce the occurrence of tissue browning in walnut explants, but the effects have been less than ideal [7–9].

Enzymatic browning is the principal cause of explant browning in plant tissue culture [4]. In generally, polyphenol oxidases (PPOs) and peroxidase (POD) are the main enzymes responsible for this browning [10]. Cells are damaged when explants are cut, and phenols are released and oxidized to quinones by enzymes, which results in enzymatic browning [11]. Furthermore, quinones formed by enzymatic reactions can form cross-links with proteins or can polymerize in tissues through a series of complex biochemical reactions, such as dehydration and polymerization, generating dark-colored melanin compounds, disrupting tissue metabolism, inhibiting growth, and, ultimately, causing browning and death of explants [12–14]. PPOs are the main enzymes leading to the oxidation of phenolic compounds [15]. Phenolic compounds are oxidized by PPOs to their quinone derivatives and further oxidized to form the pigment melanin, which is found in organisms and is responsible for browning reactions [16]. PPOs are a group of copper-containing enzymes that can catalyze the *o*-hydroxylation of monophenols to *o*-diphenols (tyrosinase activity) as well as the oxidation of *o*-diphenols to quinones (catecholase activity) in the presence of oxygen [17]. PPOs have several well-defined roles in animals, including skin/exoskeleton pigmentation and cuticle sclerotization [18]. Unlike animal and fungal PPOs, many plant PPOs lack monophenol oxidase activity. However, PPOs display rather high monophenolase activity in walnut [19]. In vivo, PPO activity is typically associated with senescent, wounded, or damaged plant tissues in which cellular compartmentalization is lost [18]. Nevertheless, the physiological role of PPOs in plants remains unclear, and most relate plant studies have focused primarily on the role of PPOs in postharvest browning, in which cut or damaged plant tissues turn brown due to the polymerization of PPO-generated quinones, which generates phytomelanin compounds [20, 21].

Walnut branches and leaves are rich in phenolic substances [22, 23], which leads to increased susceptibility to browning of generated from these tissues explants during culture [24]. Explant browning has been a difficult problem associated with walnut tissue culture for the past several decades. PPO transcription and activity play a key role in explant browning and survival. Shi [25] reported that the survival percentage of walnut scions was closely related to phenolic content and PPO activity during grafting. Araji et al. concluded that PPO plays a novel and fundamental role in secondary metabolism and acts as an indirect regulator of cell death in *J. regia* [17]. Previously, PPO was described as a single gene (*JrPPO1*) that encodes a protein with mono- and diphenolase activity in *J. regia* [26]; compared with the diphenolase activity only of PPO enzymes in other plant species, the activity of PPO in walnut is a unique feature. For many years, researchers have believed that walnut PPO activity is regulated by only a single *JrPPO1* gene, rendering it an ideal model to study enzymatic browning [17, 27]. Interestingly, *JrPPO1* appears to be constitutively expressed at a high level in all green tissues and is not responsive to wounding or methyl jasmonate treatment [26]. Previous studies have shown that walnut-bacterial blight interactions induced *JrPPO1* expression and PPO activity [28]. Using a novel purification method, Zekiri et al. purified two isoforms of JrPPO from *J. regia* leaves; these isoforms differed by a single amino acid [19]. A second homolog of *PPO* (*JrPPO2*) was discovered on the basis of the *J. regia* genome sequence, but its mechanism of action is substantially less clear [29]. Panis reported that, compared with JrPPO2, JrPPO1 exhibited a greater activity towards monophenols, whereas JrPPO2 was more active towards *o*-diphenols [30].

In the process of tissue culture, the browning rate can be reduced by optimizing the medium type and improving the culture conditions, both of which have been the focus of tissue culture research [6–9]. It has been proposed that fluid-based media, such as liquid media and semisolid media, can effectively reduce explant browning because the harmful substances in media with good fluidity can diffuse over time [31–33]. However, other studies have shown that increasing the agar (A) concentration and decreasing the fluidity can reduce explant browning, which may be related to the specific materials used [11]. In this work, walnut explants were cultured in two different types of media, namely, low-permeability agar and high-permeability vermiculite. *JrPPO* expression and phenolic content levels were determined in explants to elucidate the relationship between explant browning and *JrPPO*s and to provide new methods and theories for walnut tissue culture.

## Results

### Survival percentage of explants in different media

**Fig. 1:**
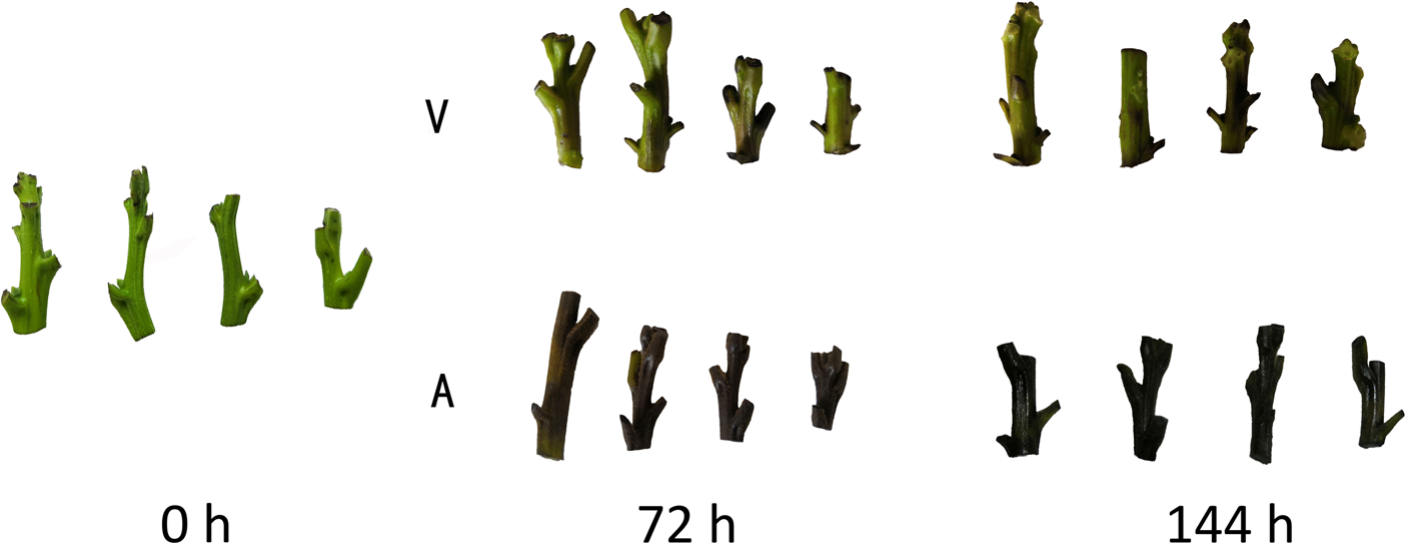
Browning of explants in different media. After 144 h, the green coloration of the explants cultured in A diminished; the explants cultured in V showed less browning, and the petiole adjacent to several buds fell off. A: agar treatment, V: vermiculite treatment

**Fig. 2:**
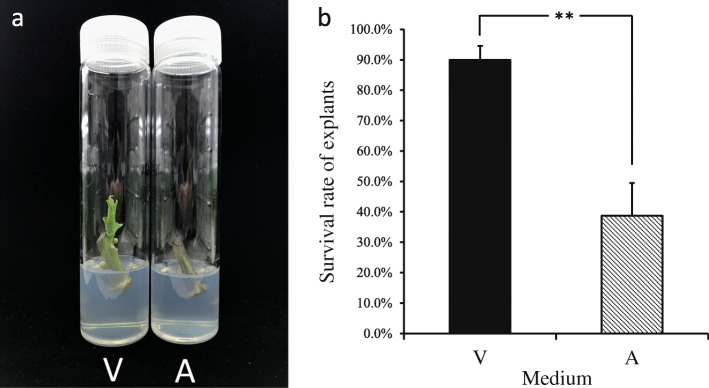
**a** Survival of explants after 14 days. **b** Percentage of surviving explants cultured in different media. The vertical bars indicate the standard deviation. The data presented in the panel are the means ± SEs. of three biological replicates. The asterisks indicate significant differences according to Student’s *t*-test: **, *P* < 0.01. A: agar treatment, V: vermiculite treatment

After 72 h of culture, the explants cultured in A exhibited severe browning, but less browning was observed the explants cultured in V. After 144 h, the green coloration of explants cultured in A diminished, and most of the media was brown; however, the explants cultured in V showed less browning, and the petioles beside several buds fell off, which indicates that the buds began to burst (Fig. 1). After 7 days of culture, the two treatment explants were transferred to fresh A-media and cultured for another 7 days to calculate the survival percentage. Compared with the those cultured in V media, the explants cultured in A, exhibited multiple areas of browning, and some buds of explants cultured in V burst and developed normally (Fig. 2a). The survival percentage of explants in A was only 38.7%, which was significantly lower than in V (89.9%) (Fig. 2b). Taken together, these results showed that initial culture in V significantly reduced the percentage of explant death and improved the survival.

### Changes in the phenolic content in explants

**Fig. 3:**
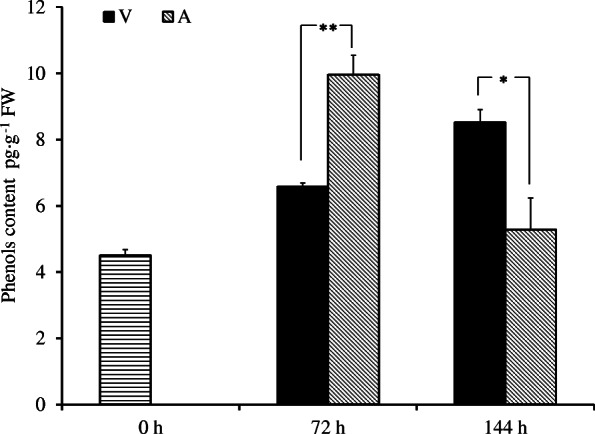
Changes in the phenol content in explants cultured in different media. Each value represents the mean ± SE of three biological replicates. The asterisks indicate significant differences according to Student’s *t*-test: *, *P* < 0.05 and **, *P* < 0.01

The changes in phenolic compounds in explants during culture in A and V are shown in Fig. 3. The phenolic content was 4.5 pg·g^− 1^ fresh weight (FW) on the day of collection, after which it increased by 121.3% after 72 h and then decreased to 5.29 pg·g^− 1^ FW in the explants cultured in A, which means that the explant phenolic content first increased but then decreased. The phenolic content in explant cultured in V was different from that in agar and increased slowly, reaching 6.57 pg·g^− 1^ FW after 72 h and 8.52 pg·g^− 1^ FW after 144 h (Fig. 3). In the early stage of culture, the phenolic content in explants cultured in A was significantly greater than that in V, but the phenolic content in explants cultured in A decreased rapidly over time and became lower than that in V. The former sterilization treatment and incision injury caused a high level of phenolic production in explants in both V and A, but the V media delayed the rapid accumulation of phenolic content. The decrease in phenolic content in A may be due to the browning-related death of explants and the decrease in metabolic activity.

### Changes in the PPO and POD activity in explants

**Fig. 4:**
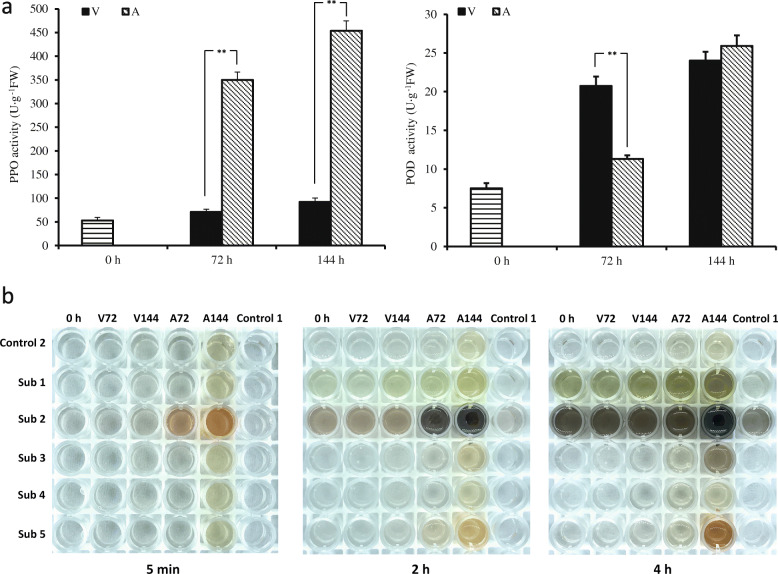
**a** Changes in PPO and POD activity in explants cultured in different media. Each value represents the mean ± S.E. of three biological replicates. The asterisks indicate significant difference according to Student’s *t*-test: **, *P* < 0.01. **b** Browning assays of JrPPO in explants together with 5 natural substrates: Sub 1 = gallic acid, Sub 2 = dopamine, Sub 3 = L-tyrosine, Sub 4 = 4-hydroxybenzoic acid, Sub 5 = protocatechuic acid. Freshly isolated protein extracts from 2 g of tissues under different treatments were incubated in the presence of substrates for the indicated time periods. The control-1 lane contains no enzyme, and the control-2 lane contains no substrates. The images were taken after 5 min, 2 h, 4 h. V72: samples from explants cultured in V for 72 h, V144: samples from explants cultured in V for 144 h, A72: samples from explants cultured in A for 72 h, A144: samples from explants cultured in A for 144 h

The PPO activity in explants cultured in V consistently remained at a relatively low level, and the PPO activity in explants cultured in A was consistently high. The PPO activity was 53.0 U·g^− 1^ FW on the day of collection. During the culture period, the PPO activity in explants cultured in V increased slightly and reached 71.0 U·g^− 1^ FW at 72 h and 92.3 U·g^− 1^ FW at 144 h. The PPO activity in explants cultured in A increased significantly and reached 349.7 U·g^− 1^ FW at 72 h and 453.4 U·g^− 1^ FW at 144 h, both of which were much greater than those of the explants cultured in V (Fig. 4a). The POD activity in explants cultured in V and A was differed. The POD activity in explants was only 7.5 U·g^− 1^ on the day of collection. The POD activity in explants cultured in V reached 20.7 U·g^− 1^ at 72 h and 24.0 U·g^− 1^at 144 h; however, the POD activity in explants cultured in A was only 11.3 U·g^− 1^at 72 h but rapidly reached 25.9 U·g^−1^ at 144 h (Fig. 4a). Therefore, the high POD activity did not cause the browning of explants cultured in V. Because POD reduces the hydrogen peroxide (H_2_O_2_)-induced increase in phenylalanine ammonia lyase (PAL) enzyme activity [34], we deduced that PPO primarily caused the browning of walnut explants, but POD slowed the rapid accumulation of phenols.

The browning assays indicated that the PPO extracted from explants exerted different activities with different substrates. All of the samples showed activity with dopamine (*o*-diphenols) after 4 h of reaction. Samples from the A treatment showed high activity with dopamine and two other *o*-diphenols (protocatechuic acid and gallic acid) especially at 144 h (Fig. 4b). However, samples from the V treatment did not react with protocatechuic acid or L-tyrosine, which indicated a low level of PPO activity. Compared with JrPPO2, JrPPO1 exerts greater activity towards monophenols and does not accept protocatechuic acid as substrate, whereas JrPPO2 is more active towards *o*-diphenols [30]. Therefore, our results suggest that there is high JrPPO2 activity under treatment A.

### Changes in *JrPPO* expression in explants cultured in different media

**Fig. 5:**
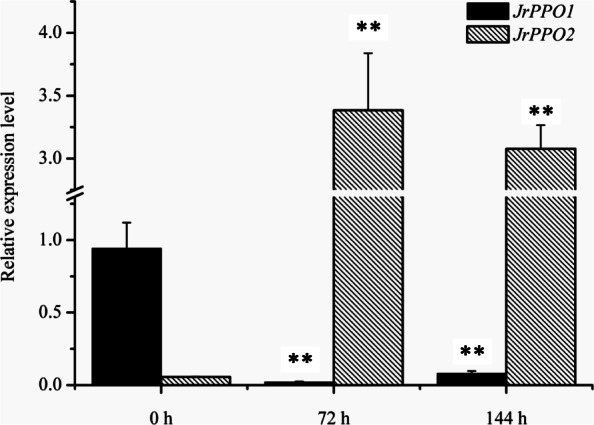
Changes in *JrPPO* expression in explants cultured in A. Each value represents the mean ± SE of six biological replicates. The asterisks indicate significant differences according to Student’s *t*-test: *, *P* < 0.05 and **, *P* < 0.01

**Fig. 6:**
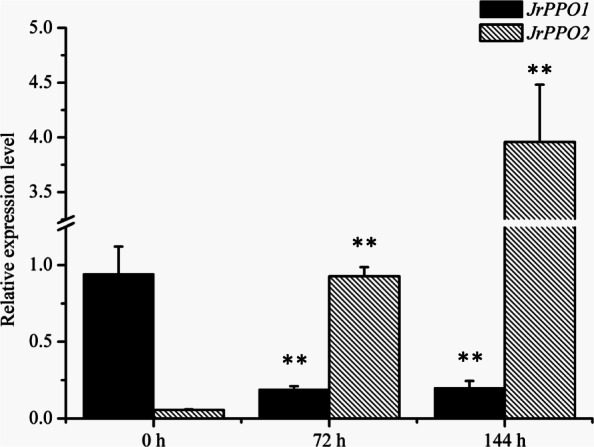
Changes in *JrPPO* expression in explants cultured in V. Each value represents the mean ± SE of six biological replicates. The asterisks indicate significant differences compared with the genes expression level at 0 h according to Student’s *t*-test: **, *P* < 0.01

Because of the obvious change in PPO activity in the explants, we cloned the PPO coding sequences (*JrPPO1* and *JrPPO2*) of walnut (Additional file 1: Fig. S1 and Additional file 2) to study gene expression patterns. Compared with the expression on day of explant collection, *JrPPO1* expression was significantly downregulated at 72 h and 144 h in the different media, whereas *JrPPO2* expression was significantly upregulated (Fig. 5 and Fig. 6). *JrPPO1* expression in the explants cultured in A was very low at both 72 h and 144 h. *JrPPO2* expression in the explants was also very low on the day of explant collection; however, the expression in the explants cultured in A increased at 72 h, but decreased slightly at 144 h (Fig. 5). *JrPPO1* expression in explants cultured in V was low at 72 h and 144 h, whereas *JrPPO2* expression in the explants cultured in V was upregulated at 144 h (Fig. 6). The relative expression of *JrPPO1* and *JrPPO2* in the explants cultured in different media exhibited opposite trends, and the trend of *JrPPO2* expression was similar to the trend of PPO activity, which suggests that *JrPPO2* plays an important role in the browning effect of phenolic compounds.

### Differences in *JrPPO* expression in different walnut tissues

**Fig. 7:**
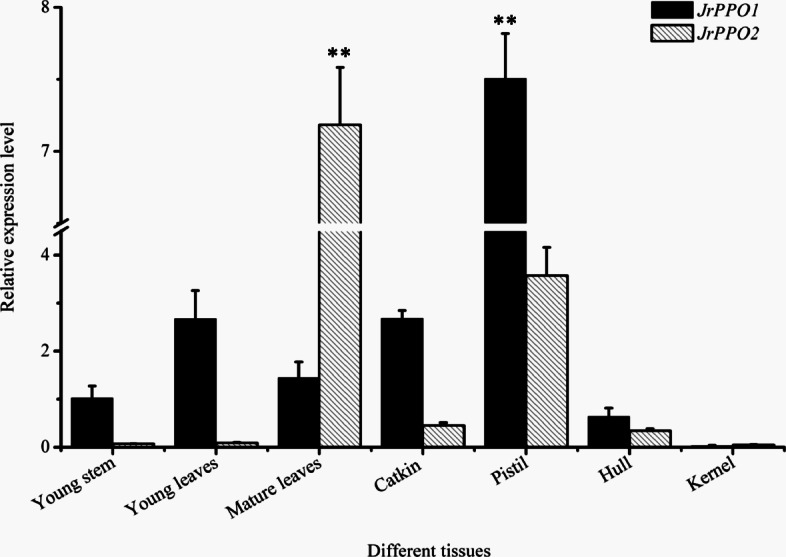
Changes in *JrPPOs* expression in different tissues. Each value represents the mean ± SE of six biological replicates. The asterisks indicate significant differences compared with the gene expression level of other tissues according to Student’s *t*-test: **, *P* < 0.01

Owing to the differences in *JrPPO1* and *JrPPO2* expression during explant browning, we analyzed the changes in the expression of these genes in different tissues from field-grown trees (Fig. 7). There were significant differences between *JrPPO1* expression and *JrPPO2* expression. *JrPPO1* was highly expressed in young stems, young leaves, mature leaves, catkins, pistils and hulls, and *JrPPO2* was highly expressed in mature leaves, catkins and pistils. *JrPPO1* and *JrPPO2* expression levels were low in kernels. The expression of the same gene also differed significantly among different tissues. The expression of *JrPPO1* was significantly greater in the pistil than in other tissues, and the expression of *JrPPO2* was significantly greater in mature leaves than in other tissues. Notably, *JrPPO1* and *JrPPO2* were highly expressed in the pistil. These results suggested that there may be differences in the division of labor between the two genes, resulting in differences in their expression in different tissues. Browning assays indicated that mature leaves, hulls and young stems showed different activities with different substrates. PPO extracted from mature leaves showed high activity with *o*-diphenols (dopamine, protocatechuic acid and gallic acid) (Additional file 3), which suggests the presence of JrPPO2, and this result is consistent with the gene expression data.

### Analysis of phylogenetic relationships of PPO in different species

**Fig. 8:**
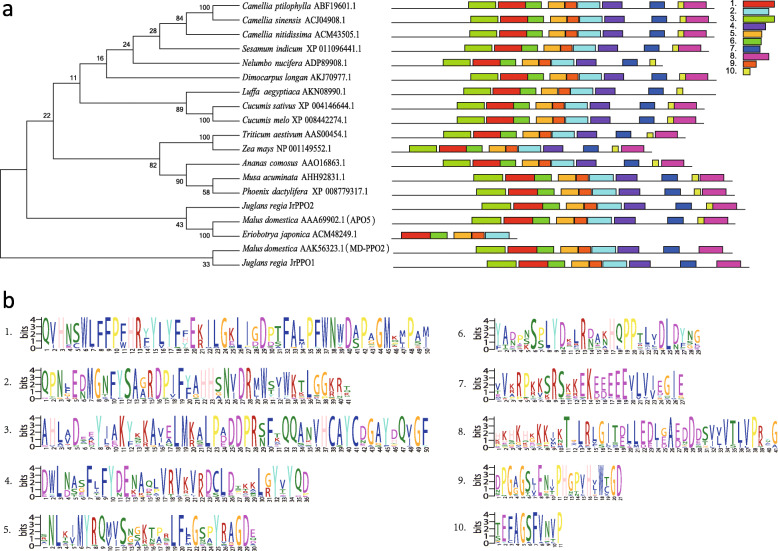
**a** Phylogeny conserved motifs of PPO proteins of 17 species. a: The motifs in the PPO proteins were identified via MEME. The boot-strap values were shown in each branch. Ten conserved motifs were identified in 17 species and shown in different colors. **b** Conserved motifs of PPO proteins of 17 species

Using MEGA 7.0, we assessed the retrieved homologous proteins along with the JrPPO1 and JrPPO2 proteins and reported sequences from 17 species (Fig. 8). Among the proteins from the 17 species, which included banana (*Musa acuminata*), date (*Phoenix dactylifera*), wheat (*Triticum aestivum*), corn (*Zea mays*) and apple (*Malus domestica*), walnut JrPPO1 was most closely related to a PPO of apple MD-PPO2 (AAK56323.1), and walnut JrPPO2 was most closely related to PPOs of apple APO5 (AAA69902.1) and loquat (ACM48249.1). The phylogenetic tree indicated that the monocotyledonous plants species, such as date, banana, pineapple, wheat and corn, grouped together, the dicotyledonous plants species grouped together, and the Rosaceae species (apple and loquat) were closely related, further indicating that PPOs have conserved throughout evolution.

We subsequently used MEME software to analyze the conservation among the sequences of these proteins. Ten conserved motifs were identified. Five common conserved motifs (motif 1, − 2, − 5, − 6 and − 9) were found among the 19 PPO proteins of the 17 species, and the distribution of conserved motifs was very similar within the same branch. With the exception of the PPOs from *Eriobotrya japonica*, *M. domestica* (MD-PPO2) and *J. regia* (JrPPO1), 16 PPOs contained conserved motifs 1–10. *M. domestica* (MD-PPO2) and *J. regia* (JrPPO1) contained conserved motifs 1–9. Motif 2 and motif 9 belong to the tyrosinase superfamily (pfam00264), which is the only member of the cl02830 superfamily. Motif 4 belongs to the PPO middle domain (pfam12142), which is the only member of the cl13563 superfamily. Motif 10 and motif 8 belong to the PPO1_KFDV domain (pfam12143), which is the only member of the cl15965 superfamily.

## Discussion

Explant browning is closely related to the phenolic content in tissue culture. Walnut explants experience stress under the environmental conditions associated with the using of cuttings and sterilization and when water and salt are present in the media [35]. Plants have established a series of defense and protection systems involving cell metabolism, hormone regulation and so on [36, 37], and the phenolic content increases to resist the impact of harsh environmental conditions, but a high accumulation of phenolic compounds leads to increased production of quinones, resulting in severe browning. In this study, walnut explants were cultured in A or V media, and a large number of phenolic compounds accumulated in the early stage, which might have been caused by damage and stress during the process of collection and sterilization. The permeability and fluidity of the media are very important to reduce explant browning [4]. The permeability and fluidity of A are low, and the harmful substances secreted by the explants during the later stage cannot be dispersed rapidly and therefore accumulate in the explants; these toxic effects result in severe browning. V is a granular and adsorptive cation-exchanger that has good permeability and fluidity; the harmful substances produced by explants can spread rapidly in V, so the browning-related death rate is low. Therefore, initial culture in V can effectively reduce the browning of walnut explants.

Plants can adapt to adversity through active and reversible regulatory mechanisms [38]. Browning is a phenomenon caused by PPO [39] and POD [40], and the levels of PPO and POD synthesis increase during plant defense responses following mechanical damage and abiotic stress [41]. PPO oxidizes phenolics to form quinones and further polymerizes quinones to form melanin compounds. However, in the presence of H_2_O_2_, POD also oxidizes phenolics and formed pigments [42]. H_2_O_2_ concentrations are very low in normal plant tissues, and there is less involvement of POD compared with PPO in browning [43]. The results of the present study showed that the POD activity of the explants cultured in V was significantly greater than that in A at 72 h, and the phenolic content in the explants cultured in V was also significantly lower than that in A. Mechanical damage to explants can induce the release of H_2_O_2_ at an early stage; especially under conditions of waterlogging stress in A. PAL activity promotes the synthesis of phenols and increases wound-induced toxicity [44]. High levels of POD enzymes rapidly degrade PAL activity caused by H_2_O_2_ release following mechanical damage [34]. The decreased PAL activity slowed the accumulation of phenolics in explants cultured in V. However, POD activity was lower at the early stage in explants cultured in A, which led to increased phenol production. Under the catalysis of highly active PPO, the phenols formed a large number of quinones, which autopolymerized to form brown-colored pigments. Therefore, the explants cultured in A were browner than the explants cultured in V. POD activity and the phenolic content in explants were greater and PPO activity was lower in V at 144 h, which caused a minimal browning of explants. Therefore, compared with POD, PPO may be the main factor that leads to the browning in walnut explants.

Plastid-localized PPO is physically separated from its phenolic substrates in intact plant cells and is distributed primarily in the vacuole. Cell damage disrupts membrane structure, and PPO oxidizes phenols to form quinones [45]. The results of Wei and Ronald showed that browning of *Pinus virginiana* Mill. inhibited callus growth, bud differentiation and rooting and was related to the accumulation of PPO [13]. The PPO activity of calli induced by buds in Scots pine (*Pinus sylvestris* L.) was significantly greater than that in immature embryos without browning [46, 47], which suggests that the high PPO activity of calli induced from mature tissue led to cell death [13] and that the increase in PPO activity in browning tissue was a response to mechanical damage and abiotic stress in these callus cultures [48]. After the explants were cut and sterilized in the present study, they were placed in A or V media, which caused further stress, and the subsequent phenolic compound content and PPO activity in the explants were significantly greater than those on the day of collection. Notably, the A media caused an increased accumulation of phenolic compounds near the wound and accelerated the browning reactions, and the activity of PPO increased continuously in the later period. The A media had poor permeability and fluidity; high levels of phenolic compounds were produced at the initial stage, and high expression of *JrPPO2* led to increased JrPPO2 activity, which resulted in the production of a large number of quinones. The metabolism in the plant tissue was disrupted, and gene expression was downregulated or abolished, which was also the reason for the high browning-related death rate of explants cultured in A media. The accumulation of phenolic compounds in the early stage was relatively low in the explants cultured in V, and *JrPPOs* expression was slowly upregulated, so these explants effectively repaired the damage during this time. Although the phenolic compound content in the explants and the expression of *JrPPO* in the later stage increased, at this time, the explants had gradually adapted to the external environment, so the browning death rate was low.

There are two *PPO* genes in apple that exhibit unique expression pattern and are associated with tissues and development [48]. *APO5* transcripts are detected only at the late stage of flower development, whereas *MD-PPO2* transcripts were was detected at all stages of flower development. In this study, *JrPPO2*, which is closely related to *APO5*, was also highly expressed in flower organs. In addition, injury resulted in significantly increased expression of *APO5* in the leaves and fruits, while *MD-PPO2* was not affected by mechanical injury [48]. The explants in this experiment were placed in media after cutting and sterilized, which caused abiotic stress, and *JrPPO2* was overexpressed, which is consistent with the expression pattern for APO5 in apple. On the basis of the evolutionary tree, APO5 and JrPPO2 are closely related, which indicates that JrPPO2 may be associated with resistance to stress. In addition, the expression patterns of *JrPPO1* and *JrPPO2* were opposite, and the change in expression of *JrPPO2* was similar to the change in PPO activity. It was thus inferred that JrPPO2 plays a key role in the oxidation of phenolic compounds. Moreover, APO5 and JrPPO2 exhibit motifs observed in other species, while both MD-PPO2 and JrPPO1 lack motif 10. It is speculated that motif 10 may function during tissue browning, but its function needs to be further studied.

## Conclusion

The rapid increase in phenolic content caused the browning and death of explants, and V media delayed the rapid accumulation of phenolic compounds in walnut explants in the short term. Therefore, the use of V as the primary culture media effectively slowed the browning of walnut shoot apical explants and improved the survival percentage. *JrPPO2* played a key role in explant browning.

## Methods

### Plant materials and treatment

Shoots (~ 10 cm long) of 6-year-old walnut (*J. regia* cv. Zanmei) trees under the same growth growing and management conditions, were collected in April from the Experimental Field of Hebei Agricultural University (EFHAU; 38°48′N; 115°24′E), China. No special permission was necessary to collect such samples. The shoots were cut into 3-cm explants with each with 1 or 2 buds. Each explant was washed in washing powder-containing water, surface-sterilized with 75% alcohol for 30 s followed by 0.1% mercuric chloride (HgCl_2_) for 8 min, rinsed 6 times in sterile distilled water, and then cultured in sterile V media [49] or A media (Table 1) in tubes. After 7 days, all the explants were transferred to fresh A media in tubes, and the death percentage of explants was determined after 14 days of culture. The cultures were maintained in a controlled environmental chamber with a 16-h light period (4 lx, provided by fluorescent bulbs) and an 8-h dark period at 25 °C. The explants were divided into two groups: one group (60 explants) for the determination of death rate; and the other group (60 explants) for RNA extraction, expression analysis and determination of phenolic contents and POD and PPO activity. Samples were collected after 0 h, 72 h and 144 h of culture. Walnut (cultivar Zanmei) tissues (shoot, young leaf, mature leaf, catkin, pistil, hulls and kernel tissues) were harvested separately from field-grown trees at the EFHAU and transported to the laboratory on ice for the PPO assays and organ-specific expression analyses. The samples were stored at − 80 °C for RNA extraction and expression analyses.

**Table 1:** Culture medium

Treatment	Media	Vermiculite(g/L)	Agar(g/L)	Sucrose(g/L)	IBA (mg/L)	6-BA (mg/L)	pH
A	DKW	0	6	30	0.01	0.75	5.5
V	DKW	600	0	30	0.01	0.75	5.5

### Evaluation of explant survival percentage

Sixty explants were further divided into 3 groups for two treatments to calculate the survival percentage after 14 days of culture (including 7 days of culture in A-DKW media). The explants for which the epidermis did not brown, the petioles fell off, and new shoots sprouted were considered to have survived. Conversely, explants for which the epidermis of the explants became brown, the petiole did not fall off, and new buds did not germinate were considered to be dead.

### PPO and POD assays

The explants were chopped into small pieces, frozen in liquid N_2_, and ground to a fine powder with a grinder. This lyophilized powder was used for activity determinations of PPO and POD enzymes and the estimation of total phenolics.

PPO was isolated from the walnut tissue as described by Higgins [26] with slight modifications. Total protein was isolated from the explants via homogenization in extraction buffer [50 mM phosphate buffer (pH 6.8), polyvinyl pyrrolidone (PVP) (1%), polyvinyl polypyrrolidone (PVPP) (2%), Triton X-100 (1%) and ascorbic acid (30 mM)]. Homogenization was performed using a 5:1 ratio of buffer (mL) to tissue (g). After homogenization, the extracts were centrifuged (12,000 rpm, 4 °C, 20 min), and the supernatant was kept at − 80 °C until use. The assay was performed as described by Anosike [50] and Mishra [51]: catechol was used as a substrate, with quinone production monitored spectrophotometrically at 420 nm (UV-3200, MAPADA, Shanghai, China). The PPO activity is reported as unit per gram of fresh explant weight.

To determine whether JrPPO1 or JrPPO2 exerted high activity in different stages or tissues, a simple browning assay was performed essentially as described by Panis [30]. Total protein (via a supernatant) was isolated from equal weights of explants or different tissues as described above. The proteins were subsequently purified as described by Zekiri [19]. Simple browning assays were performed in a reaction mixture that included sodium phosphate buffer, different substrates [monophenols (L-tyrosine, 4-hydroxyphenyzoic acid) and *o*-diphenols (gallic acid, dopamine, protocatechuic acid)] and the enzyme solution at 25 °C. One control assay was performed for each substrate in sodium phosphate buffer without enzyme solution. Another control assay was performed for each enzyme solution in sodium phosphate buffer without substrate.

POD proteins were extracted using the same method as that used to extract PPO proteins. POD activity assays were performed as described by Tian [52] and Mishra [51]: guaiacol was used as the substrate, and guaiacol polymer production was monitored spectrophotometrically at 470 nm. The POD activity is presented as units per gram of fresh explant weight.

### Total phenolics contents assays

The total phenolic content was determined according to the Folin–Ciocalteu colorimetric method [53]. Briefly, each sample (0.2 g) was ground in liquid N_2_ after which the ground sample and 15 mL of 60% ethanol were mixed together. The supernatant was collected after 90 min via ultrasonic treatment at 25 °C. The supernatant (600 μL) was subsequently added to 10 mL of distilled water, 1 mL of Folin–Ciocalteu reagent, and 2 mL of sodium carbonate (10%). After the mixture was incubated at 25 °C for 120 min, its absorbance at 765 nm was measured with a UV spectrophotometer (Shimadzu Corp., Kyoto, Japan). Gallic acid was used as a standard, and the results are expressed in milligrams of gallic acid equivalents (GAE) per gram of fresh weight. Three replicates were included in this experiment. At least 15 samples were used in each replicate.

### DNA and RNA isolation and reverse transcription polymerase chain reaction (PCR)

Young leaves were frozen in liquid N_2_ and ground to a fine powder. Genomic DNA was isolated by the use of a kit for DNA extraction from Tiangen (Product number: DP350; Tiangen, Beijing, China) to avoid phenolic interference. The explants and different tissues were frozen in liquid N_2_ and ground to a fine powder. *JrPPO1* (GenBank accession number: MW273898) and *JrPPO2* (GenBank accession number: MW273899) coding sequences (CDS) were amplified by using the primers *JrPPO1* Comp-F (5′-CCATCGATCAAGGTCCTTCATGCTTTAC − 3′), *JrPPO1* Comp-R (5′-GCTCTAGAAAATATGAAAAGGCACTGCG − 3′), *JrPPO2* Comp-F (5′-CGGAATTCGTTAACTTCGCACCAAAAGC − 3′) and *JrPPO2* Comp-R (5′-GCTCTAGATAACGTACCACAATTTCGCA-3′) and were used to analyze the *JrPPO1* and *JrPPO2* sequences respectively. Fragments were cloned into a pMDT19 vector via a TA cloning kit (Product number: 6013; Takara, Dalian, China). Vector sequencing was completed by Sangon Biotech (Shanghai) Co., Ltd. (Additional file 2).

RNA isolation of the explants and different tissues was performed using by the use of an isolation kit (Product number: DP441; Tiangen, Beijing, China). The isolated RNA was solubilized in diethyl phosphorocyanidate (DEPC)-treated RNAse-free water and the treated with DNAse. Pure RNA was used for cDNA synthesis via a commercial kit (product number: RR047A; Tiangen, Beijing, China) according to the manufacturer’s guidelines. The resulting cDNA was diluted 9-fold and stored at − 20 °C for subsequent RT-PCR and qRT-PCR assays.

### *JrPPO1* and *JrPPO2* expression analysis via quantitative real-time PCR

For gene expression quantification, specific primers were designed via Primer Premier 5.0 software for the *JrPPO1* and *JrPPO2* genes. qRT-PCR experiments were performed using an FX96 Touch™ Real-Time PCR Detection System (Bio-Rad) in conjunction with TransStart Top Green qPCR SuperMix (Product number: AQ131; TransGen Biotech, Beijing China). The primers *JrPPO1*-F (5′-CGCCAAGAACCCTACGCTAT-3′), *JrPPO1*-R (5′- GGTCCACATTCGGTCCACAT-3′), *JrPPO2*-F (5′-TGCCTTGACACCACCAAGTT-3′), and *JrPPO2*-R (5′-ACAATCGGGAAGTTGACGCT-3′) were used to analyze the *JrPPO1* and *JrPPO2* transcripts respectively. *JrACT2* (NCBI Reference: XM_018972062.1) in conjunction with specific primers (5′-TCCACCATGTTCCCTGGTAT-3′ and 5′-ACCTCCCAATCCAGACACTG-3′) was used as a reference gene for calculating the relative expression levels. The PCR mixture (20 μL) consisted of 10 μL of 2× TransStart Top Green qPCR SuperMix, 1 μL of each primer (10 μM), 1 μL of diluted cDNA and 7 μL of ddH_2_O. The following conditions were used for RT-PCR: 95 °C for 5 min; 30 cycles of 94 °C for 10 s, 63 °C for 30 s, and 72 °C for 45 s; and a final extension at 72 °C for 10 min. Three experiments (biological replicates) were performed for each sample. To normalize the total amount of cDNA present in each reaction, as an endogenous control, the walnut *JrActin* gene was coamplified. The relative expression level was calculated according to the 2^-∆∆ct^ method [54].

### Statistical analysis

The experiments were established in accordance with a completely randomized design. The data are shown as the means ± standard errors (SEs) of 3 or 6 independent biological replicates. Statistical differences between samples were analyzed via Student’s *t*-test (*P* < 0.05 or 0.01). The data were analyzed via SPSS version 20.0 (IBM Corp., Armonk, NY) and Excel 2010 software (Microsoft Corp., Redmond, WA).

### Phylogenetic tree construction

The PPO protein sequences of walnut and 16 other species were downloaded from the NCBI database (www.ncbi.nlm.nih.gov) (Additional file 4). The conserved domains of the proteins were analyzed via the Pfam protein family database (http://pfam.org/) [55] of the European Institute of Bioinformatics and the Gene Structure Display Server (GSDS; http://gsds.cbi.pku.edu.cn/) [56] and MEME (http://meme-suite.org/) websites for the PPO protein. Phylogenetic trees were constructed according to do the maximum likelihood method of MEGA 7.0 software with a bootstrap value of 1000.

## Supplementary Information

**Additional file 1: Figure. S1.** Result of *JrPPOs* CDS amplification.

**Additional file 2 **Sequencing data of *JrPPO1* and *JrPPO2.*

**Additional file 3: Figure S2.** Browning assay of JrPPO in different tissues including 5 natural substrates: Sub 1 = gallic acid, Sub 2 = dopamine, Sub 3 = L-tyrosine, Sub 4 = 4-hydroxybenzoic acid, Sub 5 = protocatechuic acid. Freshly isolated protein extracts from 2 g tissues of different tissues from filed-grown trees were incubated in the presence of substrates for time periods indicated. The control-1 lane contained no enzyme and control-2 lane contained no substrates. Photos were taken after 5 min, 2 h, 4 h. NS: samples from young stem, OL: samples from mature leaves, HU: samples from hull, KE: samples from kernel.

**Additional file 4.** Sequence alignment of PPO proteins in 17 species.

**Additional file 5.** Original images

## Data Availability

All data and materials are presented in the main paper and additional file, and sequence data generated during current study are also available in GenBank (accession numbers: MW273898 and MW273899) (https://www.ncbi.nlm.nih.gov/genbank/).

## Abbreviations

PPO: Polyphenol oxidase
POD: Peroxidase
V: Vermiculite-DKW medium
A: Agar-DKW medium
