# Supplementary figures and images for: The role of JrPPOs in the browning of walnut explants
Source: BMC Plant Biol. 2021 Jan 6;21:9. doi: 10.1186/s12870-020-02768-8 (PMC7789580; doi:10.1186/s12870-020-02768-8)

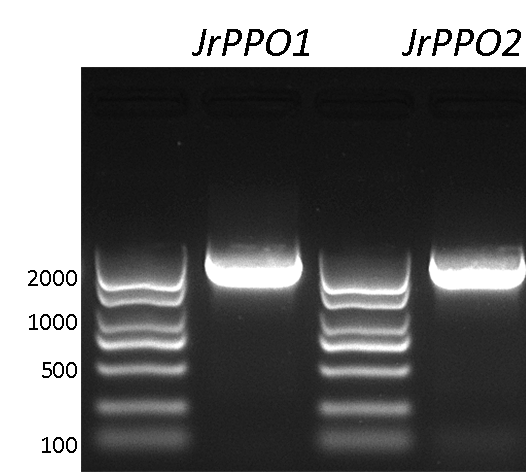

Supplement: Supplementary file 1 — Additional file 1: Figure. S1. Result of JrPPOs CDS amplification. [file 12870_2020_2768_MOESM1_ESM.tif]

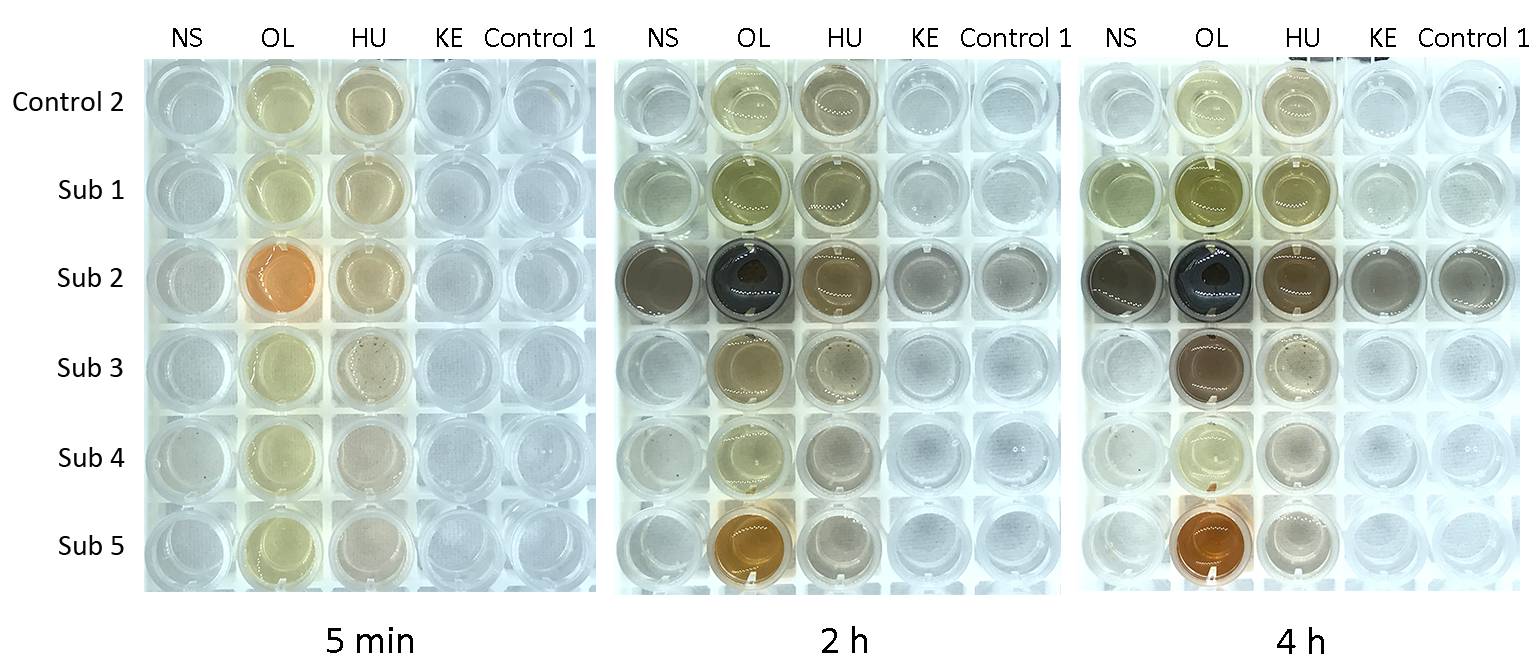

Supplement: Supplementary file 3 — Additional file 3: Figure S2. Browning assay of JrPPO in different tissues including 5 natural substrates: Sub 1 = gallic acid, Sub 2 = dopamine, Sub 3 = L-tyrosine, Sub 4 = 4-hydroxybenzoic acid, Sub 5 = protocatechuic acid. Freshly isolated protein extracts from 2 g tissues of different tissues from filed-grown trees were incubated in the presence of substrates for time periods indicated. The control-1 lane contained no enzyme and control-2 lane contained no substrates. Photos were taken after 5 min, 2 h, 4 h. NS: samples from young stem, OL: samples from mature leaves, HU: samples from hull, KE: samples from kernel. [file 12870_2020_2768_MOESM3_ESM.tif]
