# Supplementary material for: The role of JrPPOs in the browning of walnut explants
Source: BMC Plant Biol. 2021 Jan 6;21:9. doi: 10.1186/s12870-020-02768-8 (PMC7789580; doi:10.1186/s12870-020-02768-8)
Supplement: Supplementary file 5 — Additional file 5. Original images [file 12870_2020_2768_MOESM5_ESM.doc]

Original images in current study


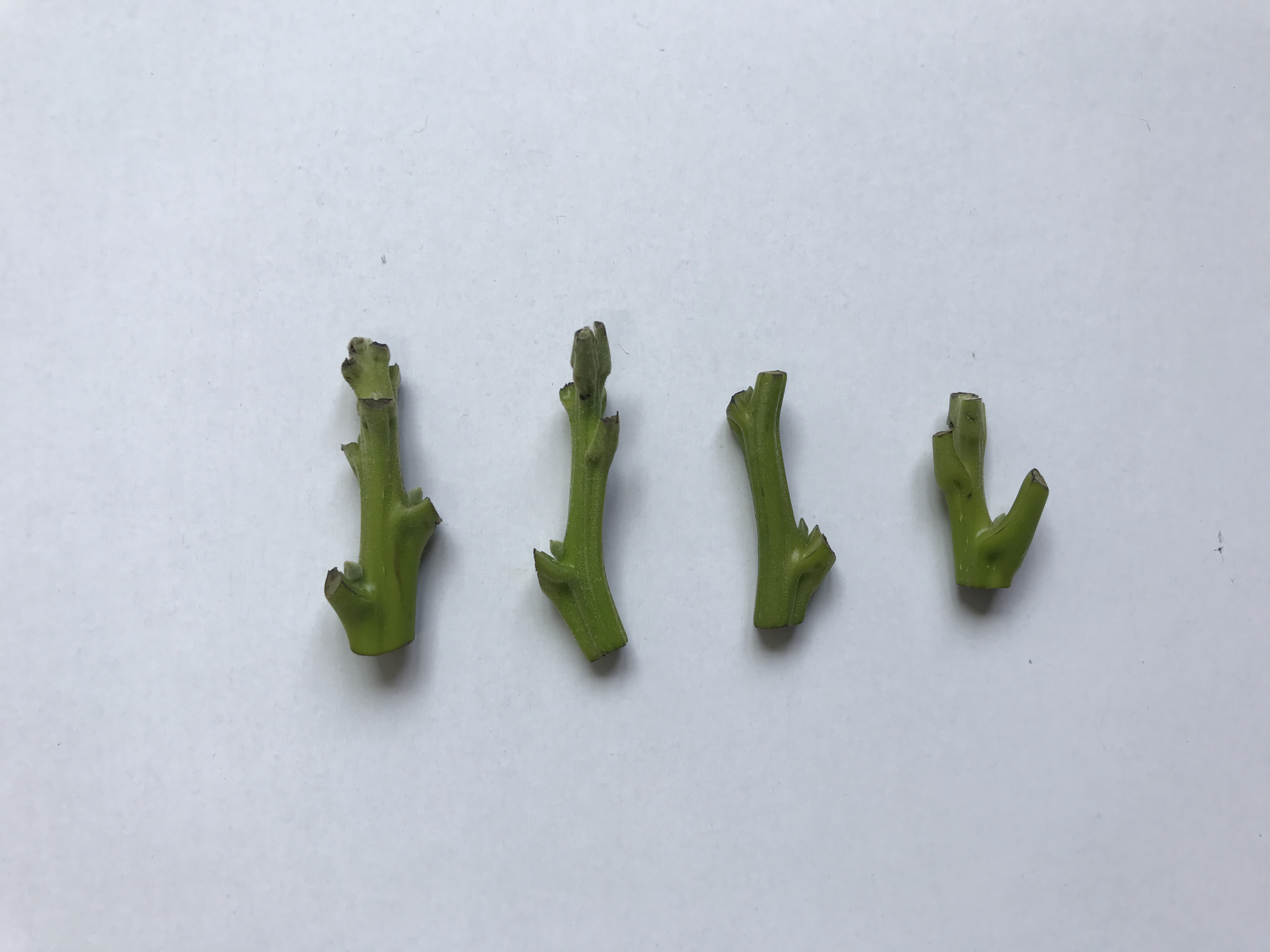


Original image Fig. 1 (0 h)
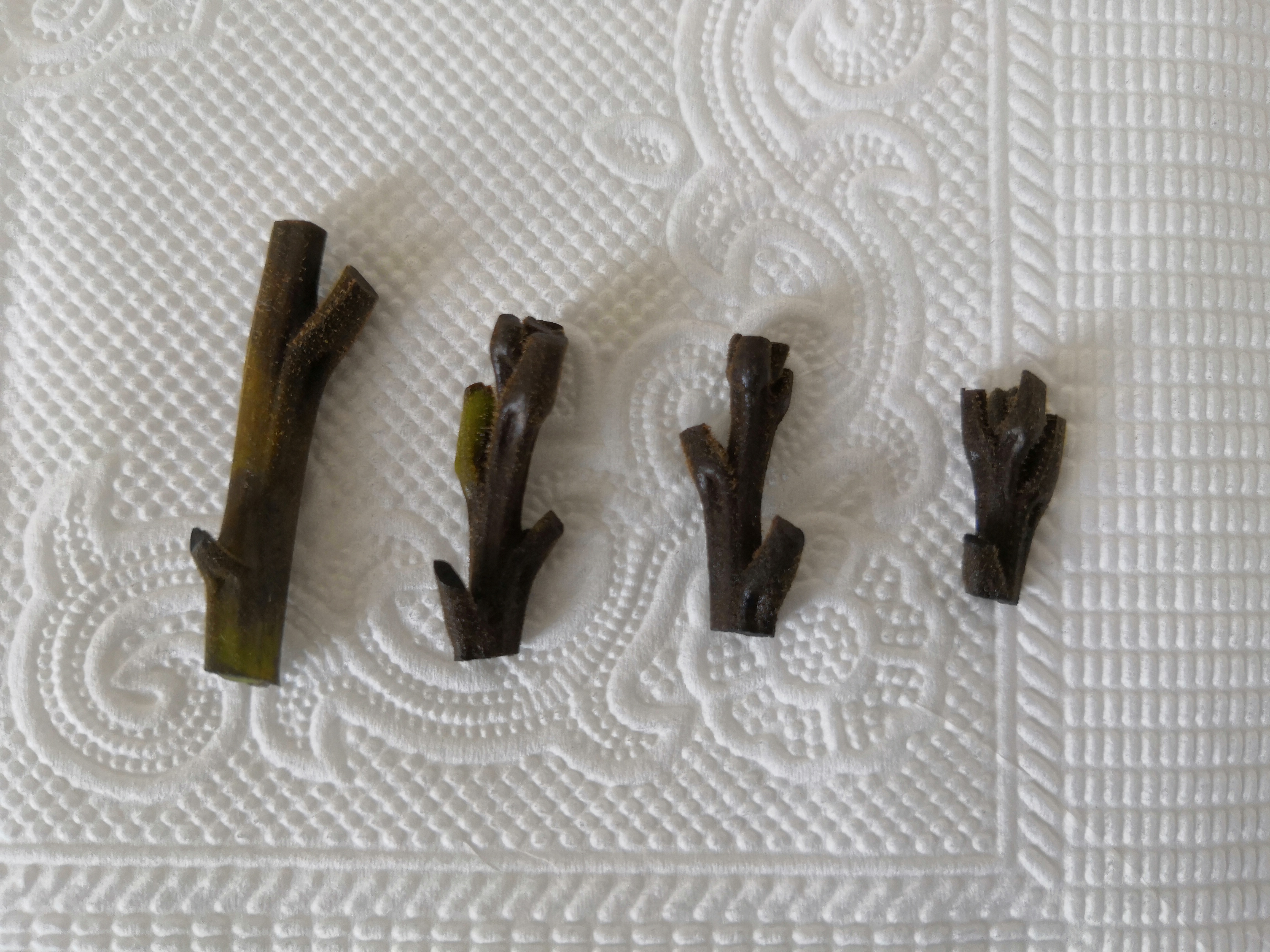


Original image Fig. 1 (A-72 h)


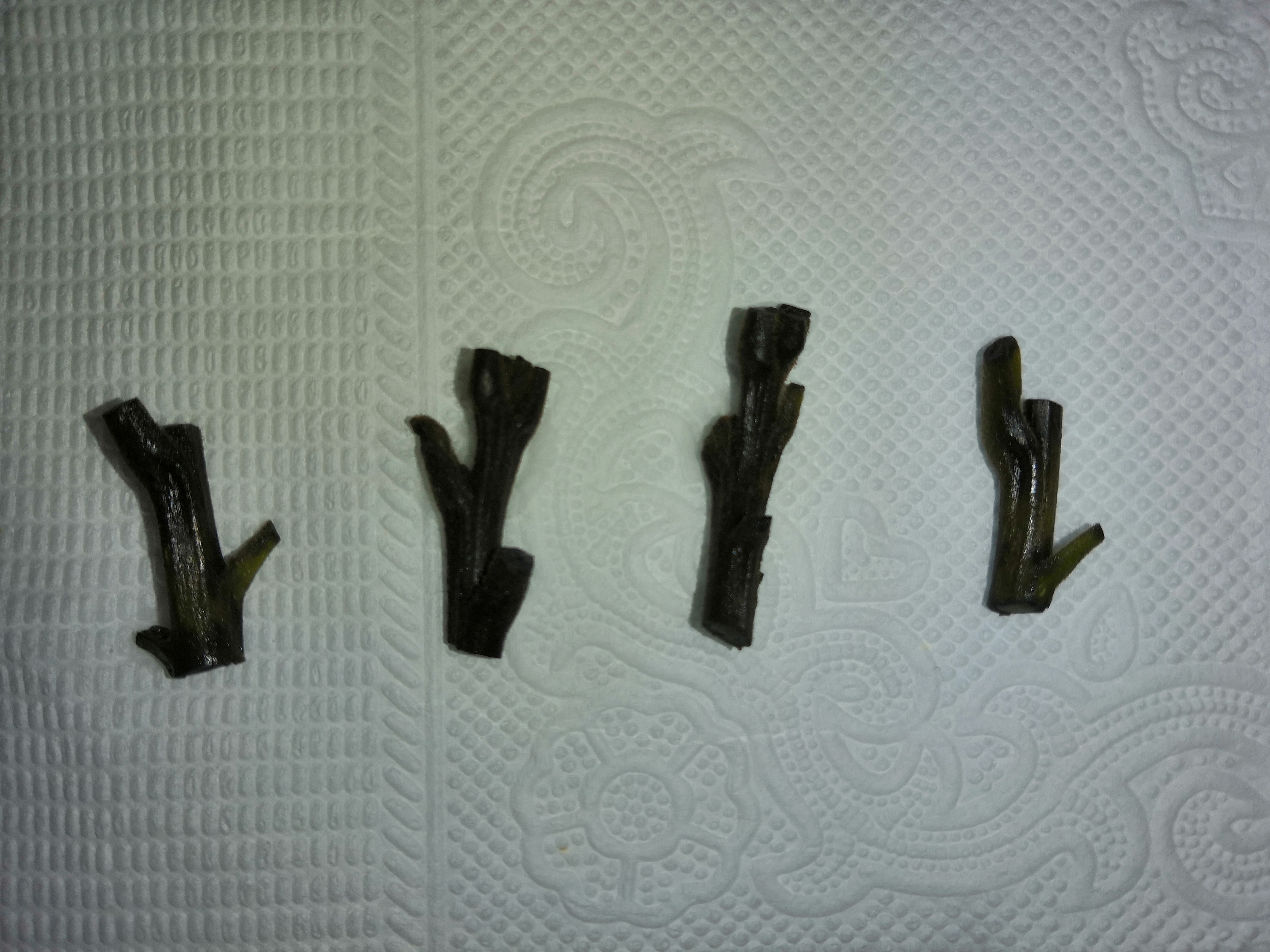


Original image Fig. 1 (A-144 h)


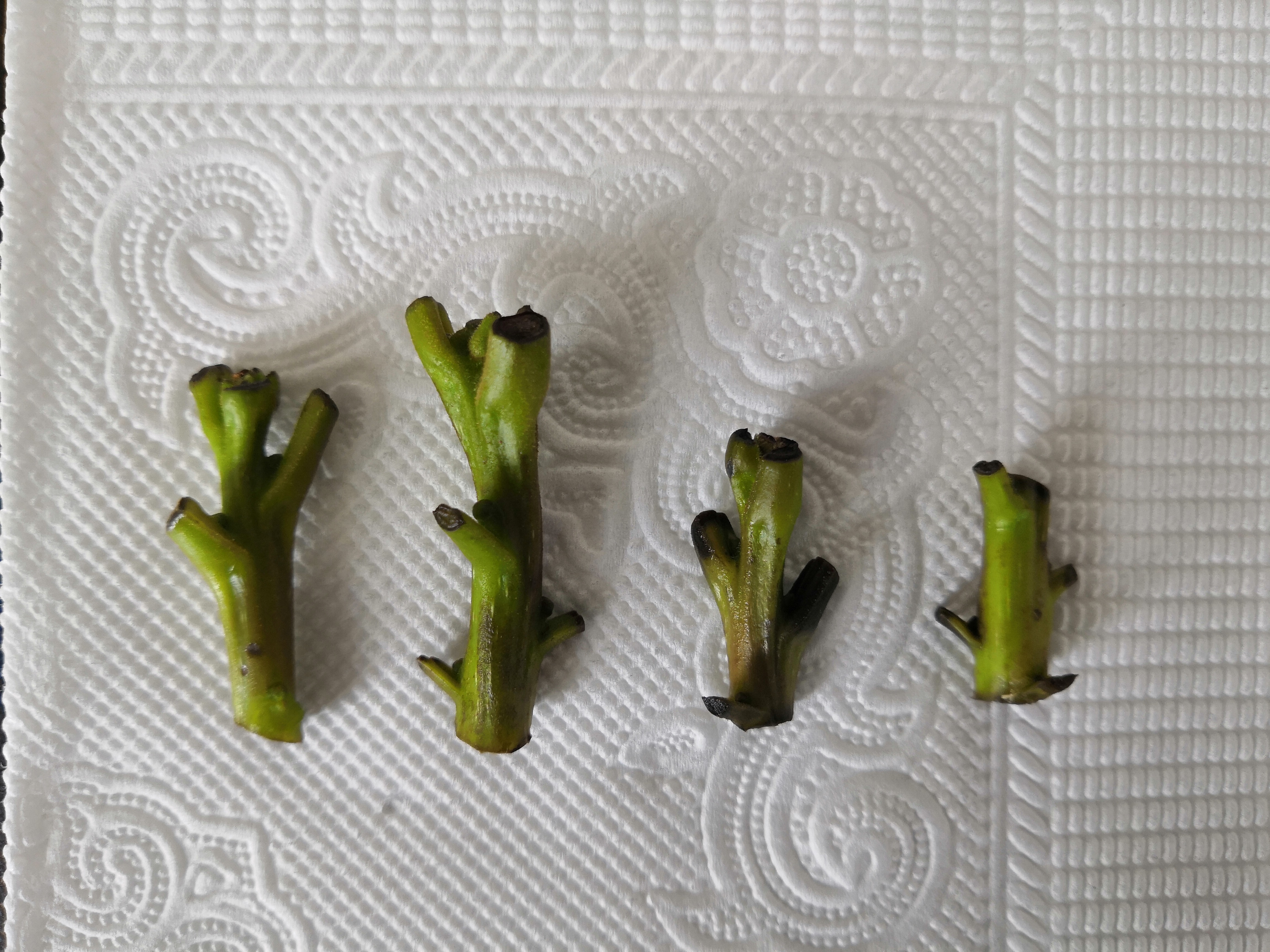


Original image Fig. 1 (V-72 h)


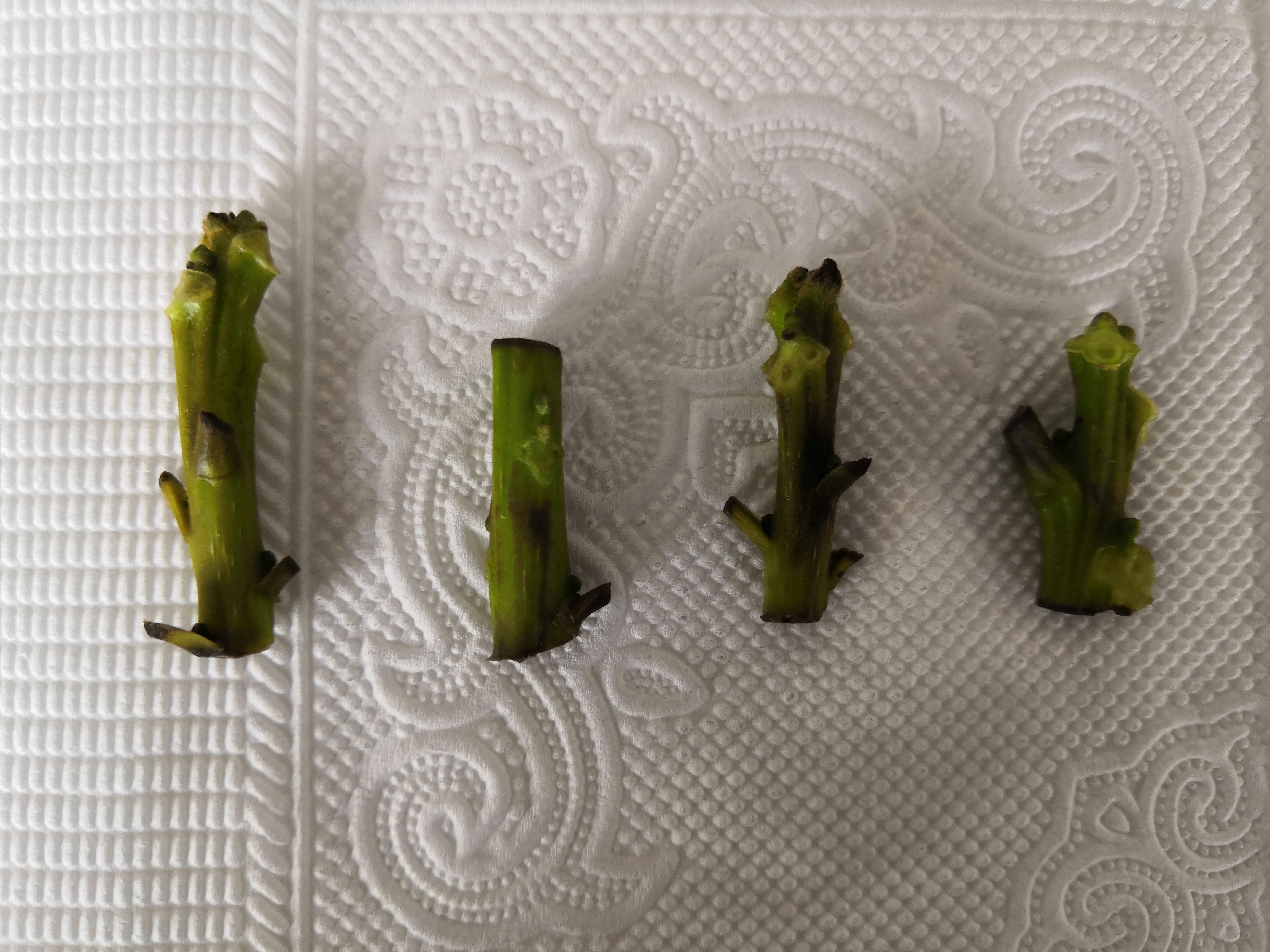


Original image Fig. 1 (V-144 h)


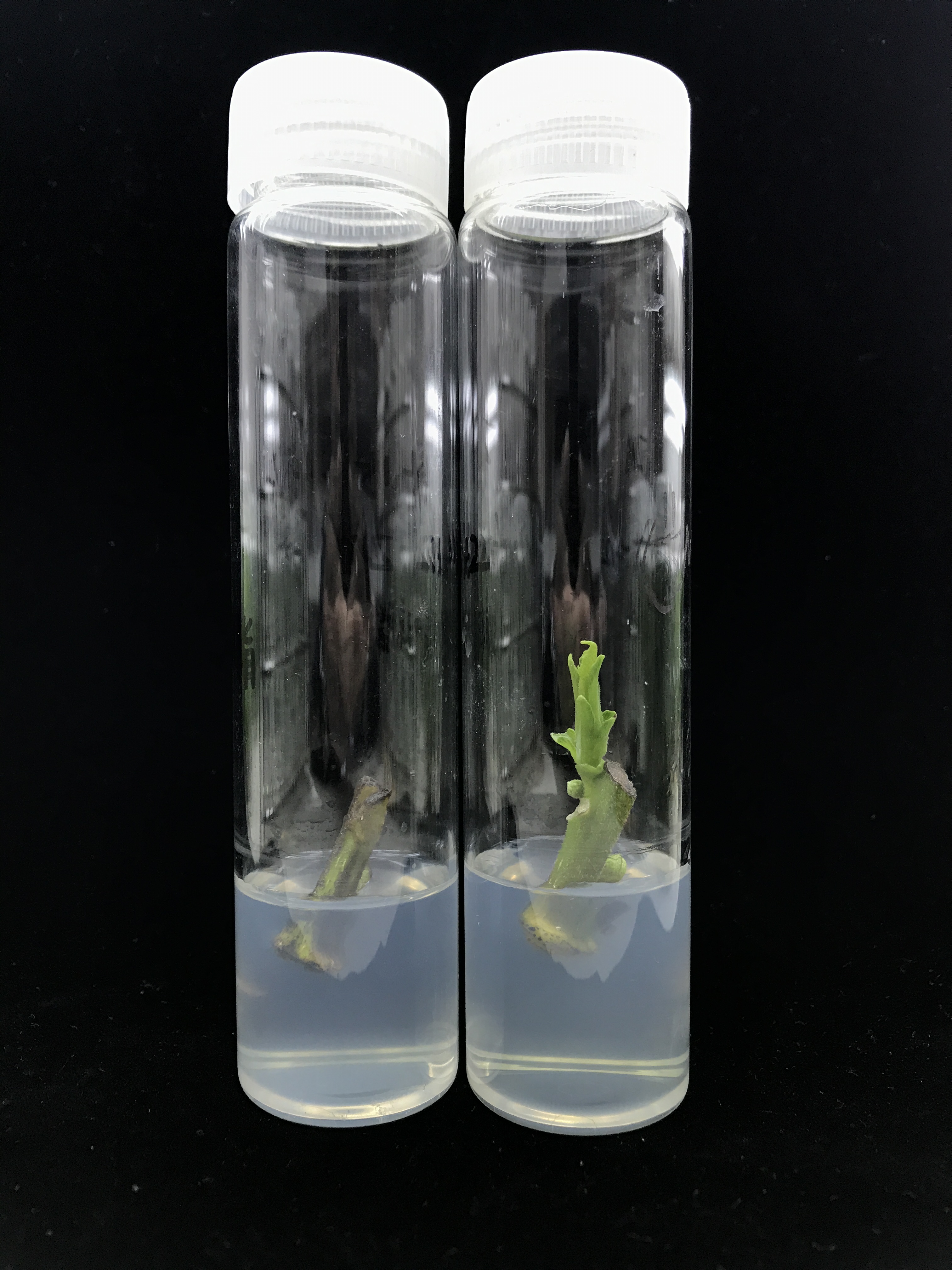


Original image Fig. 2 Survival of explants after 14 d


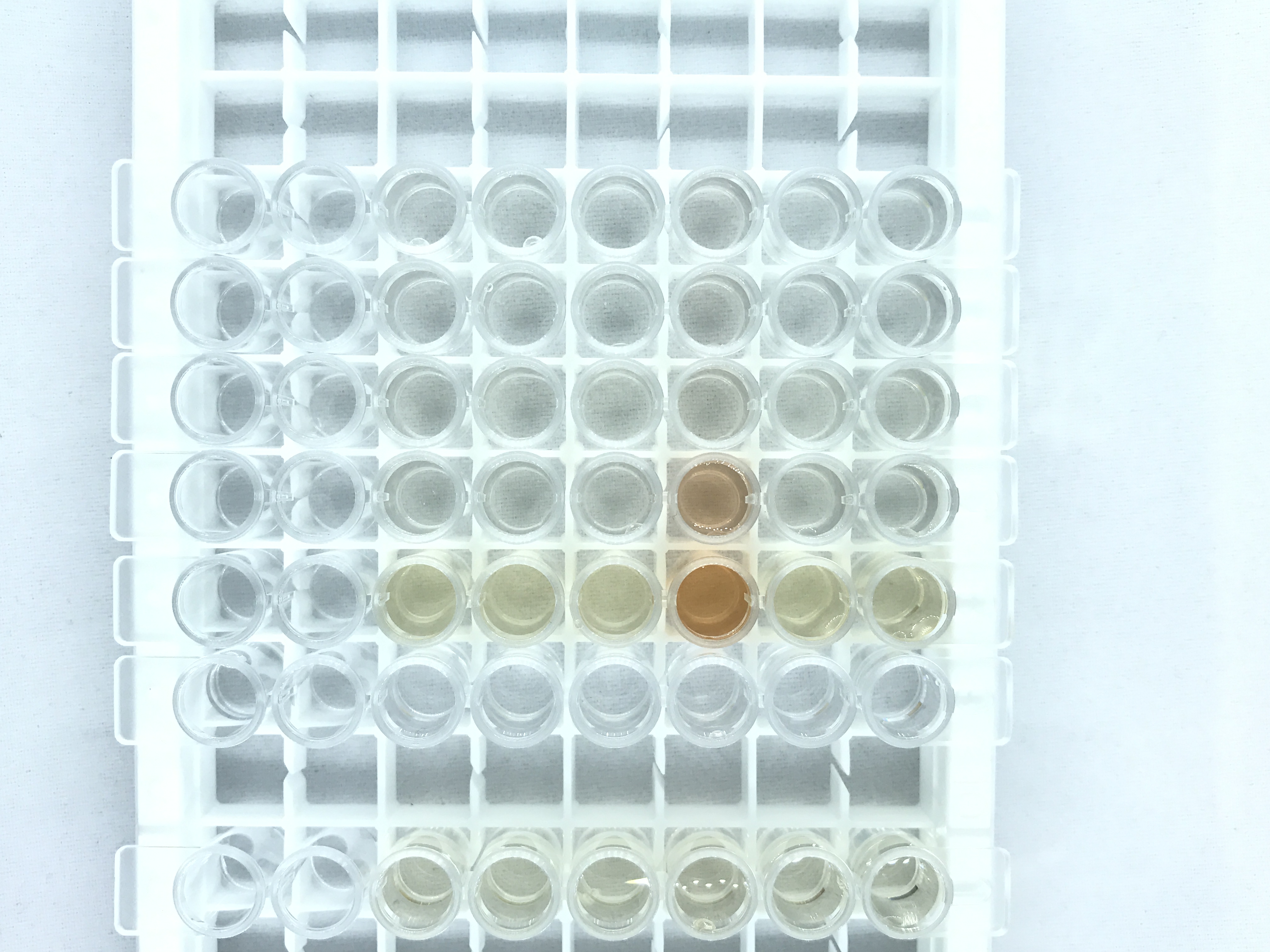


Original image Fig.4 Browning assays of JrPPO in explants (5 min)


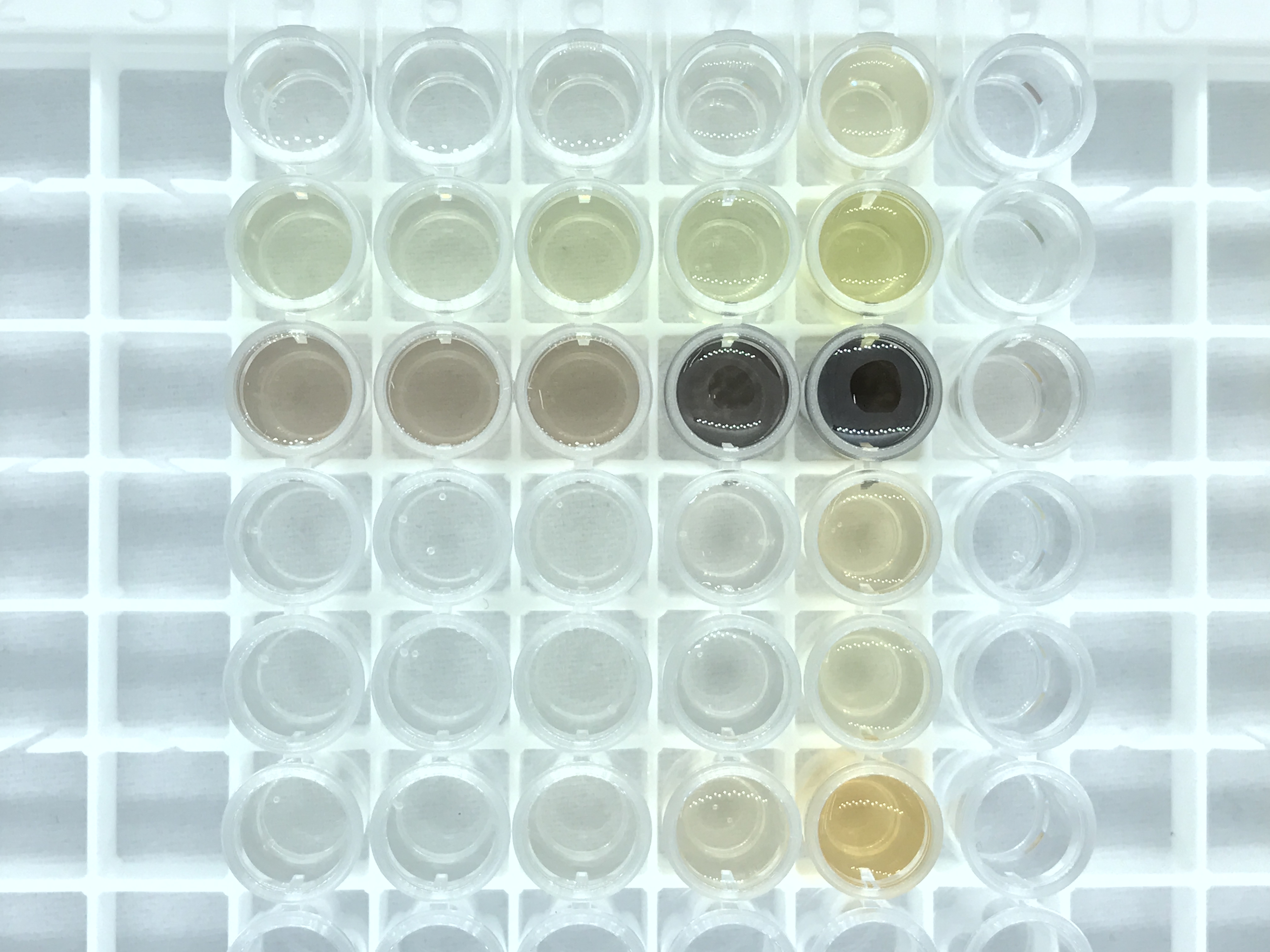


Original image Fig.4 Browning assays of JrPPO in explants (2 h)
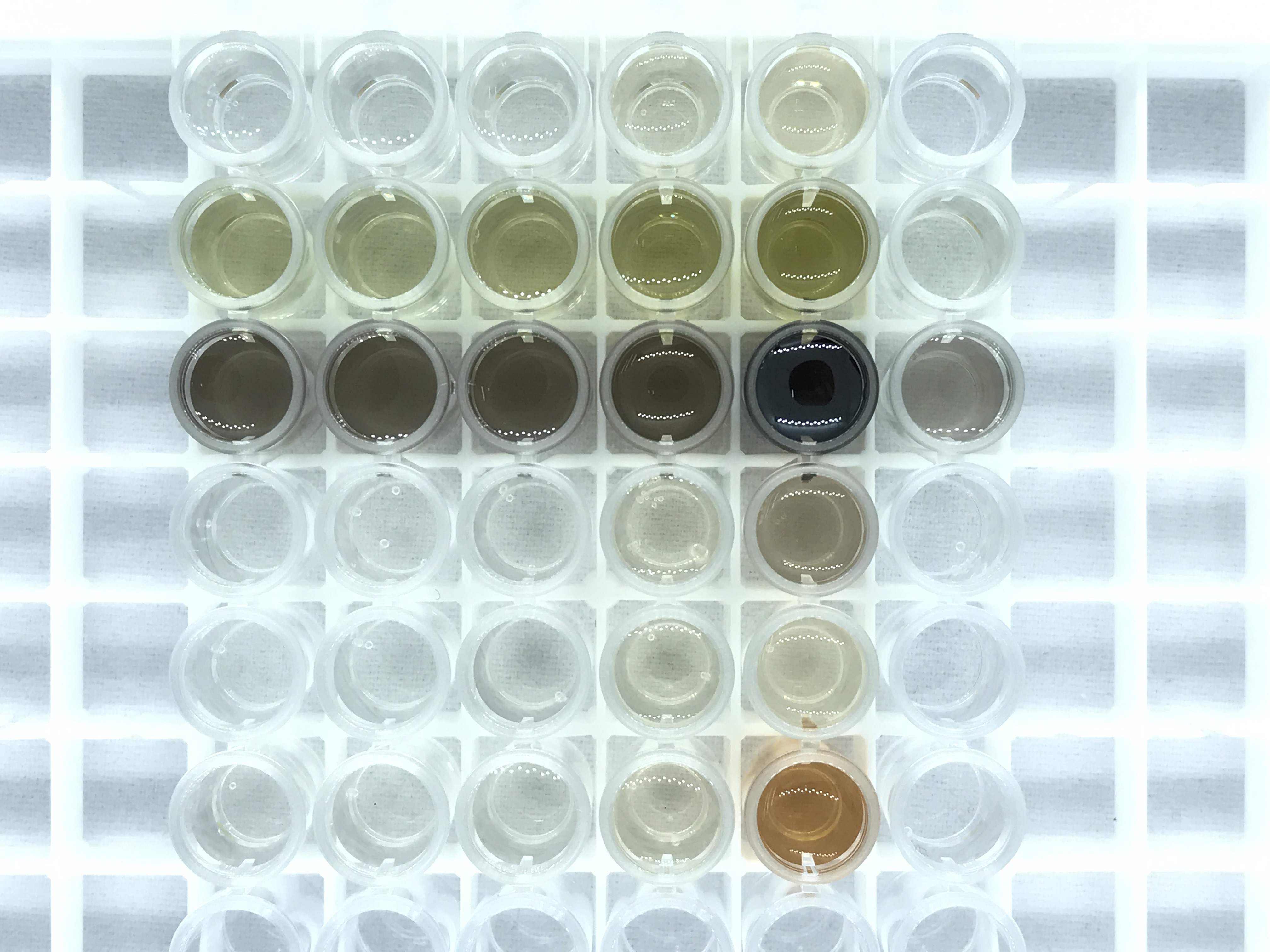


Original image Fig.4 Browning assays of JrPPO in explants (4 h)


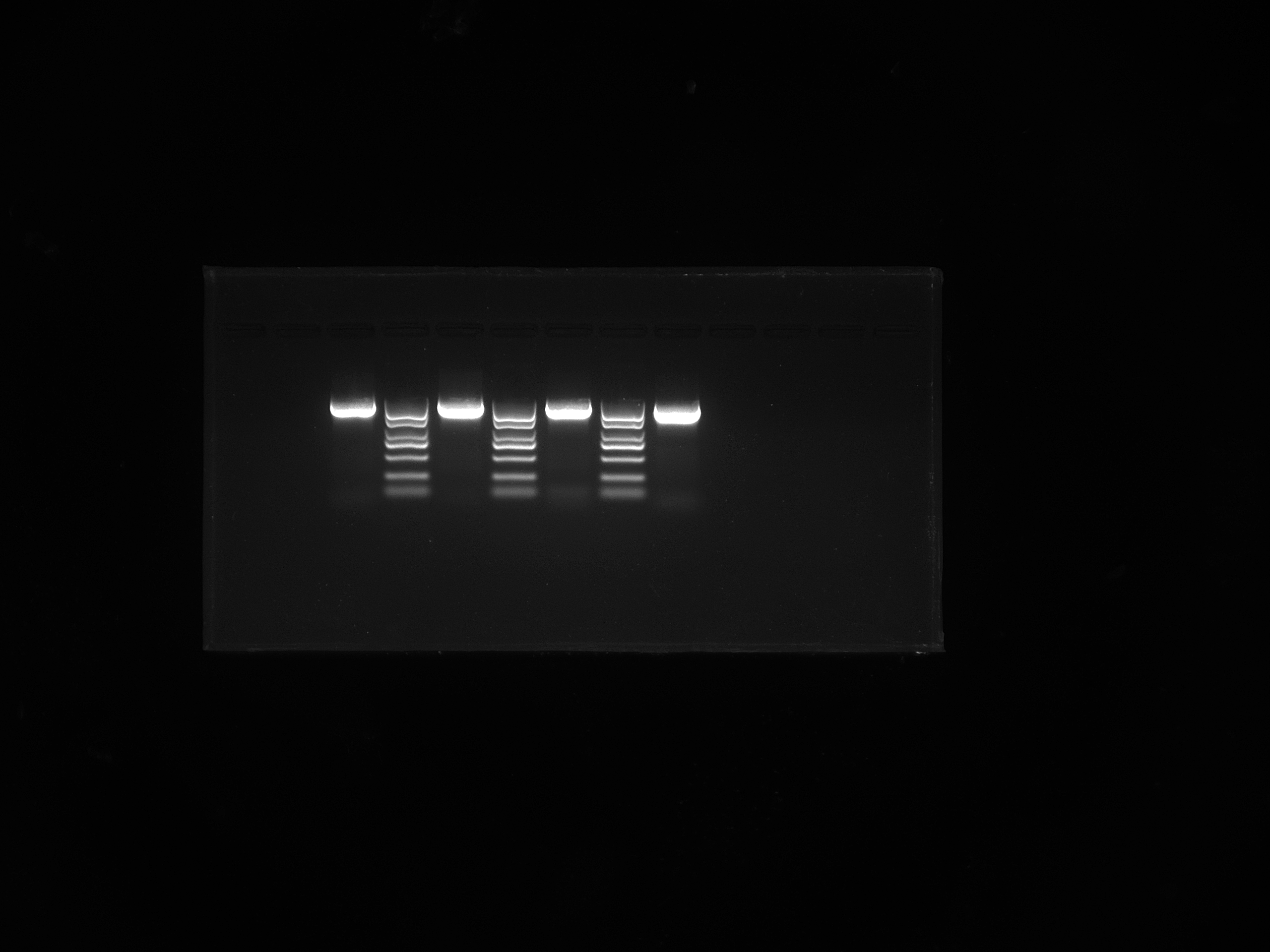


Original image of Fig. S1 Result of JrPPOs CDS amplification.

(From left to right: JrPPO1, marker, JrPPO1, marker, JrPPO2, marker, JrPPO2)


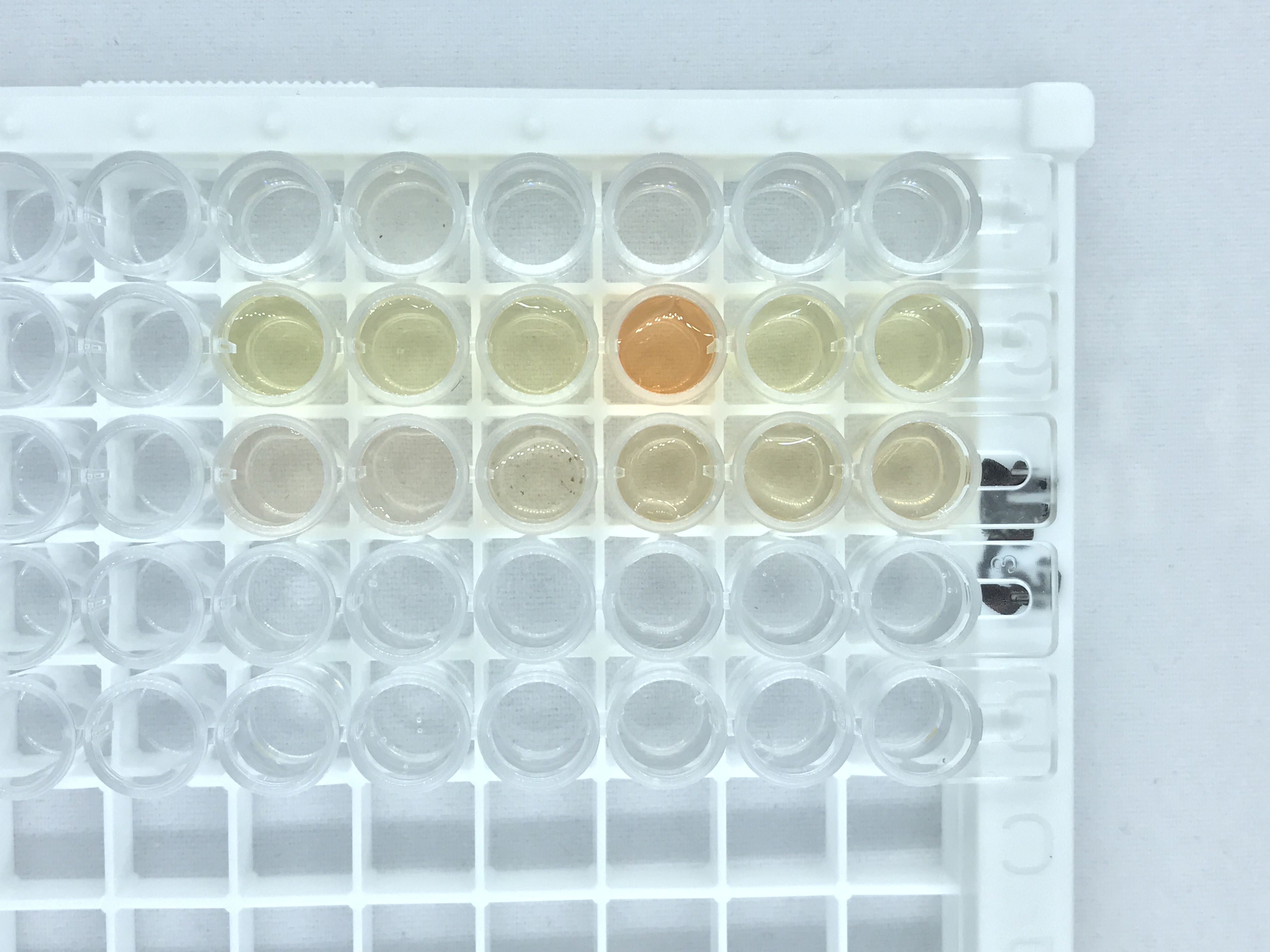


Original image Fig.S2 Browning assays of JrPPO in different tissues (5 min)


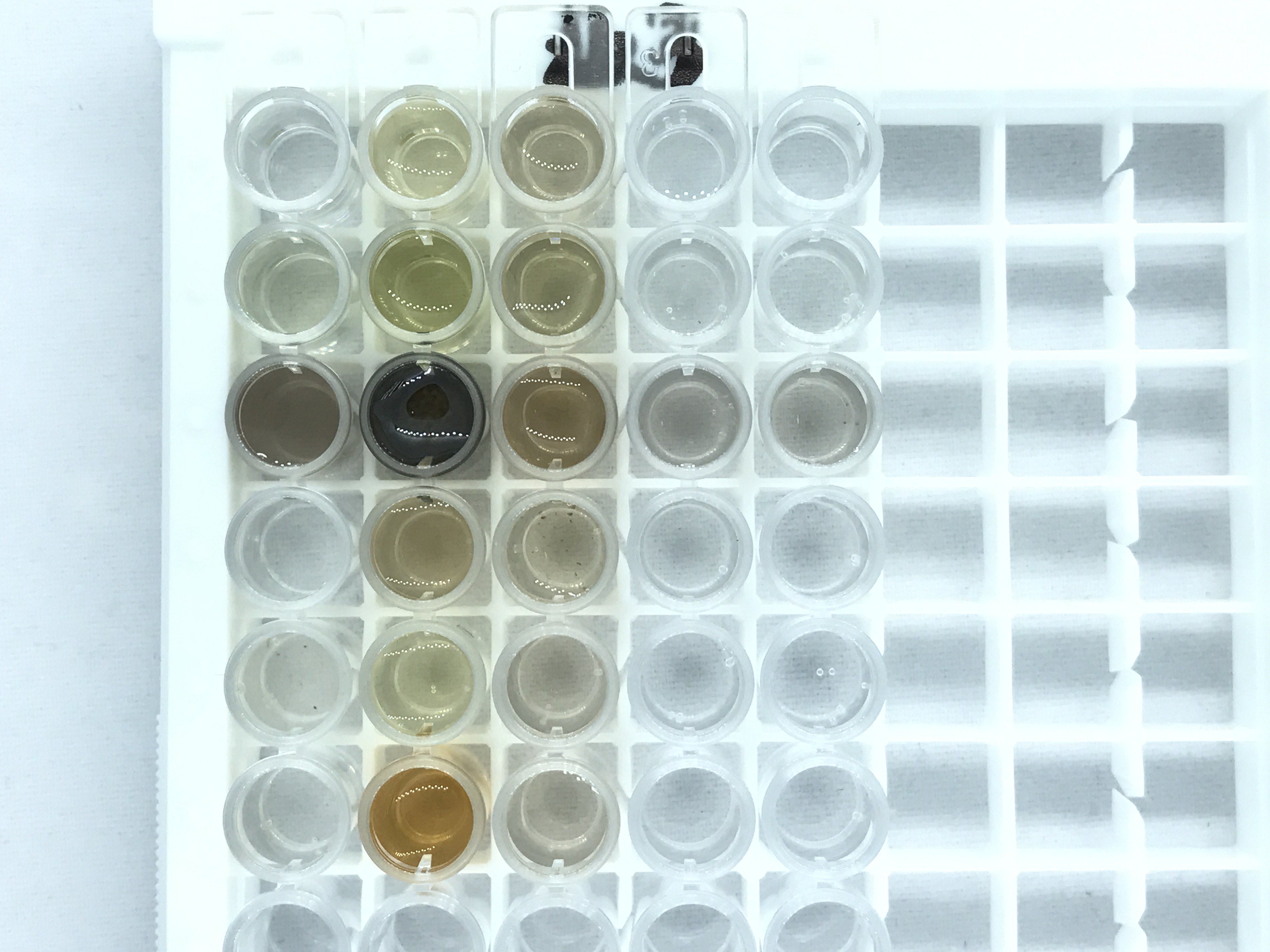


Original image Fig.S2 Browning assays of JrPPO in different tissues (2 h)


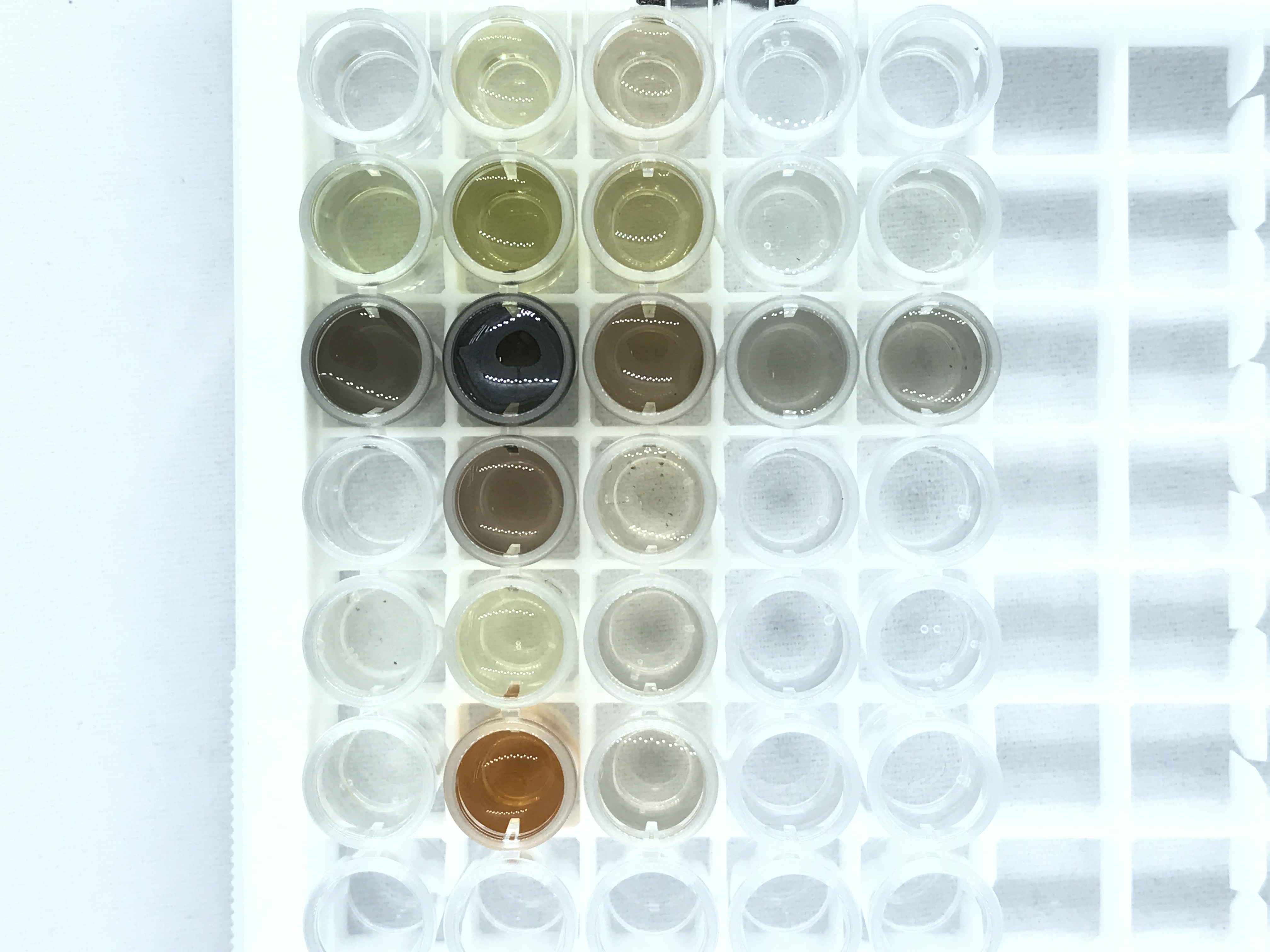


Original image Fig.S2 Browning assays of JrPPO in different tissues (4 h)
